# The process of Digitalization of Patient Education: Speeding Up During Covid-19 Pandemic

**DOI:** 10.30476/IJCBNM.2021.93265.1912

**Published:** 2022-04

**Authors:** Kolsoum Deldar, Razieh Froutan

**Affiliations:** 1 Department of Health Information Technology, School of Paramedicine, Shahroud University of Medical Sciences, Shahroud, Iran; 2 Nursing and Midwifery Care Research Center, Mashhad University of Medical Sciences, Mashhad, Iran; 3 Department of Medical Surgical Nursing, School of Nursing and Midwifery, Mashhad University of Medical Sciences, Mashhad, Iran

## DEAR EDITOR

The Coronavirus disease (COVID-19) pandemic has imposed numerous challenges on health care systems around the world, forcing them to respond quickly to this crisis. One of these challenges is the nurse-patient relationship, especially in the field of patient education. Patient education programs are conducted to empower the patients/families, improve their health outcomes, prevent disease complications and re-hospitalization, reduce healthcare costs, and improve patients’ quality of life. ^1 , 2^ However, during this pandemic, there have been several limitations such as the implementation of travel restriction or lockdown programs or even changing the public hospitals into COVID-19 centers, and the patients’ limited access to their educational programs.

In recent years, nurses changed their contact method with patients, due to  the patients’ access to medical contents on the web and their empowerment in this field. To provide better care for these online patients, nurses have tried to improve their technological knowledge and skills. For example, they began to deliver patient education contents or remotely consult with their patients through web portals, websites, mobile applications, and augmented/virtual reality technologies, or their combination. ^3^

According to the limitations induced by COVID-19 pandemic, Iranian nursing community also rapidly moved towards providing digitalized services by investing in the consumer health informatics field, specially in remote patient education programs. For example, because of early discharge of stroke patients from the stroke center to prevent contamination with coronavirus, in one project, the present authors began to use social network apps -such as WhatsApp- to continue post-discharge care and follow up at home. In this project, educational videos were sent to patients, focusing on the prevention of complications such as pressure injury. Virtual WhatsApp groups for patients and their families enabled them to share their valuable experiences with each other. ^4^ On the other hand, due to the travel limitations or lockdowns, the activity of many rehabilitation centers was also diminished, or limited to providing services to COVID-19 patients. Therefore, performing rehabilitation programs for various patients, including burn victims, resulted in serious challenges. This led to the use of approaches such as implementation of rehabilitation training programs for patients using augmented reality technology. In this project, rehabilitation exercises were taught using a pamphlet equipped with augmented reality technology. ^5^ In another project, a smart phone application was designed and implemented to teach self-care to burn patients in the areas of wound care, infection control, pain control, proper nutrition, the use of special clothing, and donor site care. ^6^

Implementing new technologies can be beneficial for many patients. They can facilitate the process of social justice in health care and strengthen and complement the traditional approaches of the health system to meet the challenges which have emerged by the COVID-19 pandemic, especially in developing and low-resources countries. Undoubtedly, despite all the problems and challenges of this crisis, it has been a great opportunity to take a closer and better look at the available facilities to improve the quality of medical and nursing care for concerned and always online patients.

## Conflict of Interest:

None declared
